# Factors Related to Nurses’ Burnout during the First Wave of Coronavirus Disease-19 in a University Hospital in Italy

**DOI:** 10.3390/ijerph18105051

**Published:** 2021-05-11

**Authors:** Francesco Bellanti, Aurelio Lo Buglio, Erika Capuano, Michał Dobrakowski, Aleksandra Kasperczyk, Sławomir Kasperczyk, Antonio Ventriglio, Gianluigi Vendemiale

**Affiliations:** 1 Department of Medical and Surgical Sciences, University of Foggia, Viale Pinto 1, 71122 Foggia, Italy; francesco.bellanti@unifg.it (F.B.); aurelio.lobuglio@unifg.it (A.L.B.); erika_capuano.556671@unifg.it (E.C.); gianluigi.vendemiale@unifg.it (G.V.); 2 Department of Biochemistry, Faculty of Medical Sciences in Zabrze, Medical University of Silesia in Katowice, Jordana 19, 41-808 Zabrze, Poland; michal.dobrakowski@poczta.fm (M.D.); olakasp@poczta.onet.pl (A.K.); kaslav@mp.pl (S.K.); 3 Department of Clinical and Experimental Medicine, University of Foggia, Viale Pinto 1, 71122 Foggia, Italy

**Keywords:** Coronavirus disease-19, burnout, nurses

## Abstract

Safety of healthcare workers in hospitals is a major concern during the COVID-19 pandemic. Being exposed for several working hours per day to infected patients, nurses dealing with COVID-19 face several issues that lead to physical/psychological breakdown. This study focused on burnout and its associated factors in nurses working in an Italian University Hospital during the first wave of COVID-19 pandemic. We designed a web-based cross-sectional study addressed to nurses working at the University Hospital in Foggia, Italy. The online questionnaire was organized in sections aimed at collecting demographic and occupational variables, including the Maslach Burnout Inventory (MBI) and the Oldenburg Burnout Inventory (OBI). Two hundred and ninety-three nurses agreed to participate. According to MBI, we reported moderate/high emotional exhaustion in 76.5%, depersonalization in 50.2%, and personal gratification in 54.6% of participants. COVID-19-related burnout measured by OBI resulted medium/high in 89.1% of participants. Among demographic and occupational factors, a multivariate regression analysis identified emotional support, consideration of leaving job, and workload as predictive of burnout in nurses. In conclusion, this study suggests that the improvement of employer and family support to nurses, as well as reduction of workload and job-related stress, would contribute to reducing burnout in nurses during COVID-19 pandemics.

## 1. Introduction

Declared as a global pandemic by the World Health Organization (WHO) in March 2020, Coronavirus disease 2019 (COVID-19) defines a spectrum of conditions sustained by the severe acute respiratory syndrome coronavirus 2 (SARS-CoV-2) ranging from mild disease to severe pneumonia [1,2]. To date, there have been 115,653,459 confirmed cases of COVID-19, including 2,571,823 deaths, reported to the WHO [3]. Italy was the first European country to be hit hard by the COVID-19 and one of the European countries registering a high number of excess deaths during the first wave of the pandemic [4].

The safety of healthcare workers in hospitals is a major concern during the COVID-19 outbreak, since more than 124,000 infections were registered by the end of March 2020 [5]. With respect to other health professionals, nurses are exposed for several working hours per day to infected patients, presenting with a higher risk of contracting the infection [6]. Hence, nurses dealing with COVID-19 are exposed to increased psychological and physical pressure, as already described in previous epidemics [7,8]. Indeed, during a pandemic, nurses experience worries about their own health, and the health of their colleagues and family [9]. Furthermore, in the context of a pandemic, nurses face several issues that lead to burnout and physical/psychological breakdown, such as perceived lack of defensive resources, rapidly changing advice about the contagion, and occupational and organizational preparedness to deal with the pandemic [9].

Burnout is defined as a protracted response to relational and emotional stressors at work, characterized by different levels of exhaustion, distrust and inadequacy, which lead to a pessimistic self-concept and work approach, with low attention for patients [10]. In particular, job burnout can be recognized as a psychological syndrome in response to working stressors: this response is constructed on three dimensions, which include exhaustion (basic individual dimension: awareness of being put under pressure and drained of both physical and emotional abilities), cynicism (interpersonal context dimension: depersonalization, disengagement from work), and reduced accomplishment (self-evaluation dimension: a feeling of ineffectiveness and lack of success at work) [10]. Both personal and work-related factors may impact burnout. 

Individual factors are identified in demographic features (age, gender, marital status), temperament, adaptive capabilities [11,12,13,14]. Work-related factors include working stress and attitude, exposition to stressful experiences, salary, social support, availability of personal protective equipment (PPE) [13,14]. Previous investigations found a high level of burnout in healthcare professionals on the front line against COVID-19, impacted by different occupational and socio-demographic factors [15,16]. In particular, the occupational factors positively associated with burnout included high workload, job stress, time pressure, and restricted support by the working organization; on the contrary, adequate PPE was reported as protective against burnout [15,16]. Very recent literature focused on the psychological risks of healthcare workers related to COVID-19 outbreak, describing high levels of anxiety, depression, stress, and burnout [17,18,19,20,21]. Working in critical situations and in a working environment characterized by a continuous contact with pain and death increases the risk of burnout for healthcare workers. Based on these theoretical premises, this study focused on the level of burnout in nurses working in an Italian University Hospital during the first wave of COVID-19 pandemic, aiming to identify associated factors. The theoretical framework underpinning our study is based on the Maslach theory of burnout, which is supported by most of the literature on burnout in nursing [22]. To identify potential predictors of burnout, we aimed attention at both demographic and occupational variables, including the working department and the exposure risk to COVID-19. We also considered the perception of risk of COVID-19 infection by nurses, and the percentage of them thinking about quitting their job.

## 2. Methods

### 2.1. Study Design and Sampling

This was designed as a web-based cross-sectional study addressed to nurses working at the University Hospital “Policlinico Riuniti” in Foggia, Italy. Data were collected from 1 June to 30 September 2020 through an online questionnaire. The study was approved by our Institutional Review Board at the Ospedali Riuniti in Foggia and performed according to the Declaration of Helsinki. Online consent was obtained from all the participants.

The sample size was estimated considering α at 0.05, a medium effect size of 0.15, power of 90%, and the number of predictors at 18 for a linear multiple regression analysis; according to these input variables, the minimum sample size required for this study was 170. Power calculation was performed with the PS Power and Sample Size Calculations, version 3. Four hundred and eight nurses were invited to complete the survey. Of all the participants, 293 (71.8%) replied to the survey with completely answered questionnaires and were used as valid data.

### 2.2. Measurements

The online questionnaire was organized in sections aimed at collecting the following information:

- Demographic variables and information regarding working Unit, professional experience, chronic diseases, previous SARS-CoV-2 infection, or contact with COVID-19 patients;
- True/false statements on the impact of COVID-19 outbreak according to factors such as organizational support, perceived risk of contracting COVID-19, workload and stress, social relationship, emotional support, perceived fatality of COVID-19, personal protective equipment, consideration of leaving the job [7];
- The Maslach Burnout Inventory–Humans Service Survey (MBI-HSS), composed of questions related to occupational burnout scored by seven level frequency ratings ranging from 0 (never) to 6 (daily). The MBI analyzes three dimensions of burnout: emotional exhaustion (EE), depersonalization (D), and personal gratification (PG). The MBI cut-off values chosen to categorize scores in low, medium, and high were as follows: for EE, low ≤ 15, medium 16–26, high ≥ 27; for D, low ≤ 8, medium 9–13, high ≥ 14; for PG, low ≥ 37, medium 36–31, high ≤ 30 (this scale is inverted; the higher the PG, the lower the burnout) [23];
- The Oldenburg Burnout Inventory (OBI), composed of 16 items related to emotional exhaustion and disengagement from work, scored by a five-point scale ranging from 1 (strongly disagree) to 5 (strongly agree). The OBI analyzes two dimensions of burnout: disengagement and exhaustion. Scores ≥ 2.1 on disengagement and ≥2.25 on exhaustion were considered as high [24].

The questionnaire included both the MBI-HSS and the OBI to improve the psychometric properties and reduce method artifacts due to one-sided questionnaire [24].

The questionnaire was translated by a mother tongue Italian health professional, and further translated back by an independent, mother-tongue English speaker who did not dispose of prior information about it. Then, the questionnaire was initially forwarded to a team of experts (two assistant professors, two associate professors, and a full professor) for internal content validity. Experts considered aims, content (demographic and occupational information, and working factors), and appropriate use of language. Then, the questionnaire was pilot tested on 20 nurses. The reliability and validity tests were applied for the pilot test, and Cronbach’s α and McDonald ω greater than 0.8 for all scales were considered as acceptable. In particular, Cronbach’s α and McDonald ω for the working factors questionnaire section were 0.87 and 0.89, respectively. 

### 2.3. Statistical Analysis

Data were expressed as count and percentages for categorical variables, and as mean ± standard deviation of the mean (SDM) for quantitative variables. Gaussian distribution of the samples was evaluated by the Kolgomorov–Smirnov test. Scale reliability and validity were assessed with Cronbach’s α and McDonald ω. Pearson’s correlation coefficient was used to correlate the MBI and OBI scores. The significance of differences was analyzed using independent *t*-tests (continuous variables, two groups), one-way analysis of variance (ANOVA) and Tukey as a post-hoc test (continuous variables, more than two groups), or in contingency tables by Pearson’s chi-square test and Fisher exact test (categorical variables). To explore factors associated with COVID-19-related burnout, multiple regression was performed using the enter method with input variables which resulted significant in the difference testing and correlation analysis. In the model, the MBI measurement was used as a dependent variable, while categorical variables such true/false statements were considered as “factors”. Finally, to test the mediating effect of working factors, a mediation analysis was performed, using the JASP version 0.14.1. All other analyses were performed using SPSS ver. 23.0 statistical software for Windows.

## 3. Results

### 3.1. Characteristics of Participants and COVID-19-Related Burnout

Demographic and occupational characteristics of nurses who filled the online questionnaire are reported in Table 1. Most participants were equally distributed between different age categories, with only 3 nurses (1%) aged more than 60 years old; 247 (84.3%) participants were women. 113 (38.6%) nurses were working in a COVID-19 Unit, and 67.5% were in an Emergency or Intensive Care Unit, or Medical Unit. More than a half of our sample was married (53.2%) or with children (58.7%), and 74.1% were not affected by any chronic disease. Of interest, even though 83.6% of nurses were exposed to COVID-19 patients, 14% got infected by the SARS-CoV-2.

Table 2 shows the number and percentage of participants who agreed with questions related to several working factors. Summarizing: (1) organizational support was considered suitable for most nurses; (2) risk perception of contracting COVID-19 was high for nurses and their relatives/friends (even though less than a half think that their contacts would be at high risk of infection); (3) workload and stress are reported by many participants, despite 30.7% mention conflicts with colleagues; (4) social relationship was mildly affected in nurses; (5) less than a half feel supported by the employer, but many report good mood at work and valorization by patients and society; (6) perceived fatality of COVID-19 was low-moderate; (7) PPE was considered efficacious and necessary/important by most participants; (8) a reduced percentage of nurses considered leaving their job.

The level of COVID-19-related burnout was assessed by two different tools, such as the MBI-HSS and the OBI, and the results are presented in Table 3. According to the MBI-HSS, a moderate/high emotional exhaustion level was reported in 76.5% of participants, while depersonalization was moderate/high in 50.2% and personal gratification was moderately/highly affected in 54.6% of cases. Among all participants, 32.4% exhibited moderate/high level of the three components of burnout. COVID-19-related burnout measured by the OBI resulted as medium/high in 89.1% of participants; exhaustion was high in 76.1% of cases, while disengagement was high in 52.2% of cases. Of interest, 94 subjects (36%) presented with moderate/high level of the three MBI components of burnout, and with medium/high burnout according to the OBI.

MBI scores were then compared with published normative values for nurses and physicians, which are 22.19, 7.12, and 36.53, for the three subscales (EE, D, PG, respectively) [25]. Of note, using one-sample t-tests to compare the current sample to the published norms cited above, our sample of nurses scored significantly higher on EE (*t* = 2.748; df = 1607; *p* = 0.061) and D subscales (*t* = 2.841; df = 1607; *p* = 0.0046), but it did not differ on PG subscale (*t* = 1.599; df = 1607; *p* = 1100).

Pearson’s correlations of the psychometric tools and their sub-dimensions are shown in Table 4.

Exhaustion detected by MBI or OBI showed a 50% concordance, with 197 (67.2%) participants presenting with a high level from both tools (Table 5).

### 3.2. Factors Associated with COVID-19-Related Burnout

Scores obtained by the MBI and the OBI were compared in participants grouped according to demographic and occupational variables, as shown in Table 6. No differences related to OBI scores were observed, while we reported differences related to exhaustion, depersonalization and gratification scores obtained through the MBI. In particular, MBI-related exhaustion score was higher in females than males, in participants presenting with at least one chronic disease than those without, and in nurses working for more than 20 years as compared to those working 1–5 years; moreover, MBI-related depersonalization score was higher in participants infected by SARS-CoV-2 with respect to not-infected ones; finally, the MBI-related gratification score was lower in nurses working in COVID-19 Units than those engaged in COVID-19-free Units, and in nurses working in other departments than emergency departments.

**Figure 1:**
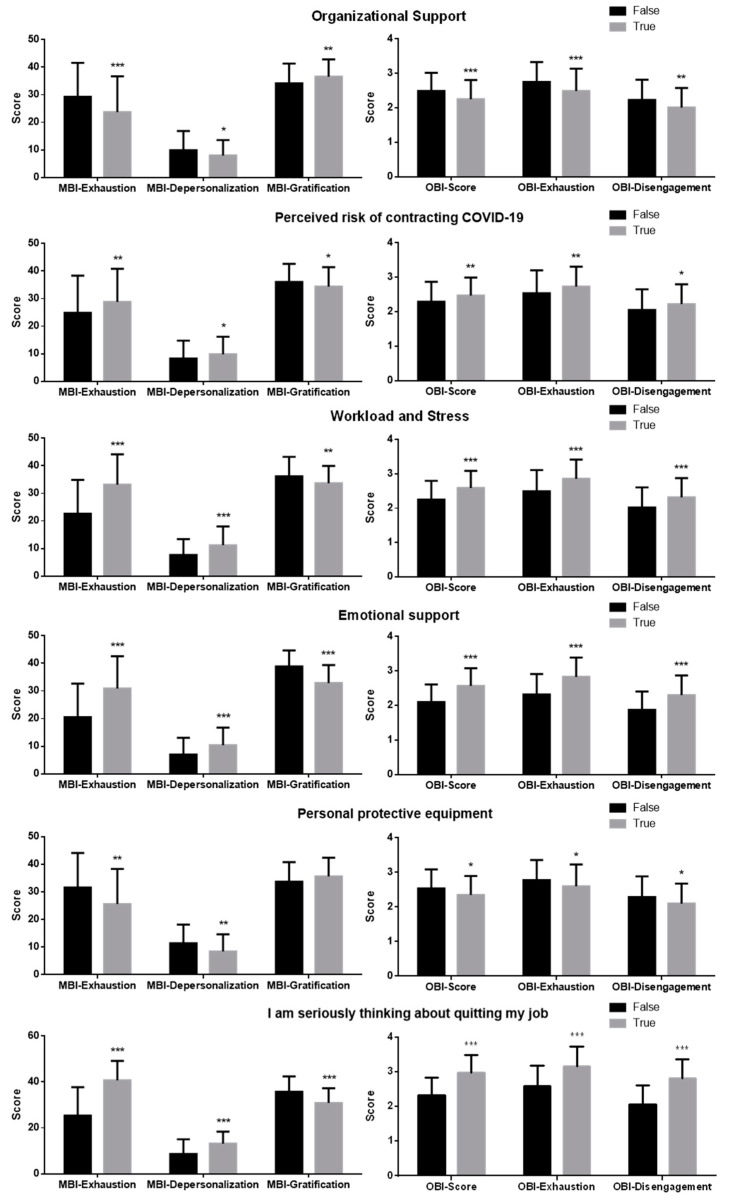
Comparison of scores obtained by the Maslach Burnout Inventory (MBI) and the Oldenburg Burnout Inventory (OBI) in participants grouped according to the working factors reported in the graph titles. Data are expressed as mean ± standard deviation. Statistical differences were assessed by independent student’s *t*-test. * = *p* < 0.05; ** = *p* < 0.01; *** = *p* < 0.001.

We then analyzed scores obtained by the MBI and the OBI in participants referring to answers provided to statements related to working factors (Figure 1). Nurses who agreed with most of the statements related to the same factor were grouped and compared with the group who disagreed. Participants who disagreed with measures of organizational support and PPE showed higher MBI- and OBI-related scores (apart from MBI-related gratification score, which was lower), as compared to those who agreed. Furthermore, scores were greater (and gratification was lower) in nurses who agreed with statements related to a perceived risk of contracting COVID-19, workload and stress, and emotional support, with respect to nurses who disagreed. Of interest, all the scores were higher (with lower gratification) in nurses which agreed with the statement “I am seriously thinking about quitting my job”, as compared to those who disagreed.

**Figure 2:**
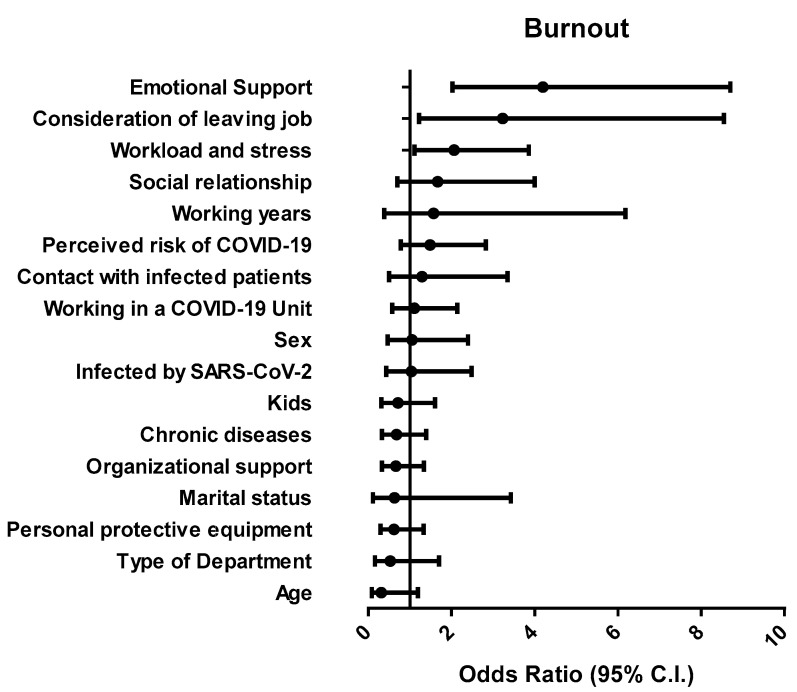
Forest plot showing adjusted odds ratio for the frequency distribution analysis of burnout.

Univariate analysis showed that factors associated with burnout (detected by both questionnaires) in nurses were consideration of leaving the job (F = 23.809, *p* < 0.001), workload and stress (F = 12.567, *p* < 0.001), and emotional support (F = 33.812, *p* < 0.001), while factors associated with exhaustion were consideration of leaving the job (F = 27.928, *p* < 0.001), workload and stress (F = 38.718, *p* < 0.001), social relationship (F = 4.577, *p* = 0.033), emotional support (F = 33.082, *p* < 0.001), working in a COVID-19 Unit (F = 5.182, *p* = 0.024), and having kids (F = 6.544, *p* = 0.011). To identify potential predictors of burnout, a multivariate regression model was created, that considered both demographic and occupational factors. The model was significant (chi-square = 82.916, *p* < 0.001) and the proportion of variance explained ranged from 24.6% (Cox and Snell) to 34.4% (Nagelkerke). As shown in Figure 2, factors such as emotional support (β = 1.457; *p* < 0.001), consideration of leaving job (β = 1.306; *p* = 0.018), and workload (β = 0.753; *p* = 0.023) were predictive of burnout in nurses (Figure 2).

Finally, the results of the mediation analysis are shown in Table 7. A direct effect of workload and stress on exhaustion, of social relationship on depersonalization, and of emotional support on both exhaustion and personal gratification were estimated as significant.

## 4. Discussion

The COVID-19 pandemic caused an overall rise of psychological problems, including anxiety, depressive disorders, insomnia, and burnout in health care workers [17]. This study highlighted the psychological impact of the first wave of COVID-19 on nurses working in an Italian University hospital. Levels of burnout and exhaustion were consistent, since we found that almost 90% of participants met the criteria for medium/high burnout, while almost 70% exhibited emotional exhaustion.

Burnout is one of the most important determinants for discomfort and well-being alterations in health professionals. A consensus has recently defined burnout as an occupational, physical, and emotional exhaustion associated with continued exposure to work-related issues [26]. Indeed, burnout causes reduced commitment to job-related activities, de-personalization, and decreased working abilities [27]. Causes of burnout are frequently distinguished in individual and work-related variables [28]. The present study focused on the identification of determinant factors of burnout in nurses to provide information necessary to reduce and prevent it during the ensuing waves of COVID-19 outbreak. To this, we adapted scales from previous investigations which measured burnout in other pandemics [7,8]. Psychometric properties of both MBI-HSS and OBI were previously reviewed, showing satisfactory content validity, structural validity, and internal consistency [29]. Pandemics exert a significant psychological and emotional impact on nurses, who are indispensable to the healthcare support [30]. Indeed, pandemics worsen the stress perceived by nurses, since they face strong physical, cognitive, and emotional demands [31,32]. According to mortality data, the first wave of the COVID-19 pandemic in Italy started on February 2020 and was considered as ended in the second half of May 2020 [33]. Even though less than 40% of participants were working in COVID-19 Units, 83.4% cared for COVID-19 patients, and 14% got infected by SARS-CoV-2. A small but significant proportion of nurses (16%) considered they should not care for COVID-19 patients, and 12.9% of participants were thinking about being transferred to different Units or quitting their job. 

A previous study analyzed burnout in frontline nurses during the first wave of pandemics in Wuhan, finding that about half of the participants reported moderate/high levels according to the MBI [34]. Another study used the MBI in nurses during the first wave of pandemics in Japan, finding an overall burnout prevalence of 31.4% [35]. In our study, we used the OBI in addition to the MBI, identifying burnout in nurses presenting with high scores according to both tools. Of note, the convergent validity of both instruments has been previously studied [24]. Our results showed a 36% prevalence of burnout, according to both questionnaires. Among all the burnout sub-domains analyzed by both MBI and OBI, exhaustion was reported in more than 70% of nurses, with a 50% concordance between tests. A study performed on Italian nurses before the pandemic estimated moderate/high-frequency level of 47.5% for exhaustion, 54.7% for depersonalization, and 57.3% for personal unsatisfaction [36]. Results from another Italian study suggested that working with COVID-19 patients and in areas with high rates of contagion is associated with high levels of stress, burnout, secondary trauma, anxiety, and depression in healthcare professionals [19]. Even though we could not directly compare our results with previous data, the present study strongly suggests that the COVID-19 outbreak increased the frequency of burnout in nurses.

To address the causes of burnout in nurses during the first wave of COVID-19 pandemics, we considered several demographic and occupational factors, as well as seven working-related factors as potential predictors. 

Of note, emotional support, consideration of leaving job, and workload and stress were predictive of burnout. Fears about personal safety and wellbeing of the family have been previously described as determinant factors for burnout related to COVID-19 [37]. The COVID-19 pandemics caused a sudden rise in hospital admissions, with a consequent impact of workload and stress in nurses. A previous study suggested that each extra patient added to the workload of a nurse was associated with a 23% increase in burnout [38]. On the contrary, we do not retain that shift work was a factor related to burnout, since no differences were reported in burnout-related scores between nurses working in different departments. Interestingly, exposure to patients infected by COVID-19 or working in COVID-19 department did not result as predictive factors for burnout, differently from previous studies [8,19,39]. Stress is a factor significantly influencing burnout in several studies [11,12]. This study indicates that work-related stress induced by the first wave of COVID-19 pandemics is a factor associated with burnout in nurses. Thus, during the spread of an emerging infectious disease, management of workload and stress would contribute to prevent or reduce nurses’ burnout.

Other than workload and stress, employer consideration and support from family and friends were also predictive of burnout in nurses. Indeed, attention by the employer and assistance from colleagues or family were described as protecting effects that may directly or indirectly reduce burnout [13,14]. Thus, these aspects should be considered for further research in future investigations.

It is worth noting that factors significantly linked to burnout (workload and stress, consideration of leaving the job, and emotional support) were predictive of burnout even before the pandemic effect of COVID-19 [13,14,38]. On the contrary, factors directly related to COVID-19 (such as perceived risk or fatality of the disease) did not result significantly associated to burnout. A possible explanation of this apparently paradoxical result may be offered by the impact of pandemic on motivating the dedication of nurses, increasing their empathy for patients, as well as providing a sense of satisfaction, with consequent decrease of burnout-related dimensions. Taken together, the results of this study indicate that the COVID-19 pandemics led to an exacerbation of the ongoing underlying problems that were pre-existing workplace factors contributing to nurse burnout. The final message of this investigation synergizes with similar studies in promoting immediate interventions for the management of psychological issues in the workplace [17,18,19,20,21].

Limitations of our study include the recruitment of a small number of participants from a single hospital in Italy, so that these findings cannot be generalizable to other regions or countries. Moreover, data obtained by self-administered questionnaires were not related to clinical data on healthcare professionals’ health. Further, work factors considered in the study were assessed as true or false statements rather than on a Likert-type scale, so that correlation or linear regression analysis could not be performed. The relatively small variance explained by the significant factors, moderate concordance between tests examining similar concepts, and the possible influences of the large number of variables in the regression model represent additional limitations of this investigation. Finally, levels of burnout were not measured before the COVID-19 pandemics; thus, no comparisons on prevalence changes were possible.

## 5. Conclusions

A high proportion of nurses presented with burnout—mostly characterized by exhaustion—during the first wave of COVID-19 pandemics. Our data showed that burnout in nurses was not dependent on demographic characteristics or occupational factors such as working in a COVID-19 department or being directly exposed to infected patients, but it was associated with emotional support, consideration of leaving job, workload, and stress. These results lead to important theoretical and practical meanings as they indicate that the COVID-19 pandemic exerts a negative impact on nurses’ well-being. The outcome of this investigation provides basic information aimed at contributing to programs and strategies for the reduction of burnout in nurses. To this, hospital managers should focus on the improvement of their valorization to nurses, on the promotion of support by family and society, and on the reduction of extra work. Furthermore, investments on mental wellbeing strategies and psychological interventions are encouraged to improve the healthcare of nurses during possible future pandemics.

## Figures and Tables

**Table 1:** Demographic and occupational characteristics of the studied population.

	*N* (%)
**Age (years):**	
21–30	69 (23.5)
31–40	85 (29.0)
41–50	75 (25.6)
51–60	61 (20.8)
>60	3 (1.0)
**Sex:**	
M	46 (15.7)
F	247 (84.3)
**Working in a COVID-19 Unit:**	113 (38.6)
**Type of Hospital Unit:**	
Emergency and Intensive Care	81 (27.6)
Medical	117 (39.9)
Surgical	50 (17.1)
Diagnostic Services	45 (15.4)
**Years of Service:**	
1–5	81 (27.6)
6–10	45 (14.3)
11–15	41 (14.0)
16–20	30 (10.2)
>20	99 (33.8)
**Married:**	156 (53.2)
**With children:**	172 (58.7)
**Chronic diseases:**	
None	217 (74.1)
1	57 (19.5)
2	16 (5.5)
3	3 (1.0)
**Infected by SARS-CoV-2:**	41 (14.0)
**Exposed to COVID-19 patients:**	245 (83.6)

**Table 2:** Impact of COVID-19 outbreak according to the analyzed working factors.

Factor	Question	N. of “True” (%)
Organizational support	I have someone to turn to when I face a problem in using personal protective equipment	211 (72.0)
	Support is available to workers who need help	167 (57.0)
	Clear protocols were established for everyone	166 (56.7)
	Most staff adhered to the recommended measures	253 (86.3)
	I found easy to comply with the recommended measures	209 (71.3)
	The staff in my Unit is appropriate	136 (46.4)
Perceived risk of contracting COVID-19	My job exposes me to a high risk of contracting the SARS-CoV-2	256 (87.4)
	I am afraid of being infected	217 (74.1)
	I cannot perceive the risk of infection	37 (12.6)
	My family is worried for me	225 (76.8)
	I think that people close to me are at high risk of infection because of my job	136 (46.4)
	People close to me are worried for my health	249 (85.0)
	People close to me are afraid of getting infected because of me	122 (41.6)
Workload and stress	Conflict among colleagues is increased in the last 3 months	90 (30.7)
	I feel more stressed at work	229 (78.2)
	My workload has increased	246 (84.0)
	I must work overtime	174 (59.4)
	I must do things that I should not do at work	151 (51.5)
Social relationship	It was not easy to tell my family about the risk I am exposed to	102 (34.2)
	People avoid me because of my job	55 (18.8)
	People avoid my family because of my job	28 (9.6)
	I avoid telling people about my job nowadays	71 (24.2)
Emotional support	I am sure that my employer will care for me in case I get COVID-19	134 (45.7)
	I feel valued by my employer	94 (32.1)
	I feel valued by patients and by society because of my job	180 (61.4)
	The mood is good at work	213 (72.7)
Perceived fatality of COVID-19	If I get COVID-19, I will not survive	21 (7.2)
	I think my risk of dying of COVID-19 (over the next year) is higher than dying from a road traffic accident	122 (41.6)
	I think my risk of dying of COVID-19 (over the next year) is higher than dying of cancer	125 (42.7)
Personal protective equipment	Personal protective equipment at work is efficacious	228 (77.8)
	I am fully persuaded of the necessity and importance of personal protective equipment	289 (98.6)
Consideration of leaving the job	I should not take care of COVID-19 patients	47 (16.0)
	I am trying to be transferred in a different Unit or to find another job because of COVID-19 risk	8 (2.7)
	I am seriously thinking about quitting my job	30 (10.2)

**Table 3:** Results from the Maslach Burnout Inventory (MBI) and the Oldenburg Burnout Inventory (OBI).

MBI Scores	Range Interval	Mean	SDM
Emotional Exhaustion	0–54	26.95	12.89
Depersonalization	0–28	9.09	6.46
Personal Gratification	16–48	35.20	6.87
**MBI (*n*, %)**	**Low**	**Moderate**	**High**
Emotional Exhaustion	69 (23.5)	70 (23.9)	154 (52.6)
Depersonalization	146 (49.8)	77 (26.3)	70 (23.9)
Personal Gratification	133 (45.4)	88 (30.0)	72 (24.6)
**OBI Scores**	**Range Interval**	**Mean**	**SDM**
COVID-19-related burnout	1.06–3.01	2.39	0.56
Exhaustion	1.00–4.00	2.64	0.62
Disengagement	1.00–3.88	2.14	0.59
**OBI (*n*, %)**	**Low**	**Medium**	**High**
COVID-19-related burnout	32 (10.9)	168 (57.4)	93 (31.7)
Exhaustion	70 (23.9)		223 (76.1)
Disengagement	140 (47.8)		153 (52.2)

Values in round brackets indicate row percentages. SDM, standard deviation of the mean.

**Table 4:** Pearson’s correlation of the OBI scores and the MBI dimensions.

	OBI Exhaustion	OBI Disengagement	OBI Score
MBI Exhaustion			
*r*	0.716	0.665	0.754
*p*	<0.001	<0.001	<0.001
*N*	293	293	293
MBI Depersonalization			
*r*	0.384	0.550	0.508
*p*	<0.001	<0.001	<0.001
*N*	293	293	293
MBI Gratification			
*r*	−0.453	−0.505	−0.523
*p*	<0.001	<0.001	<0.001
*N*	293	293	293

**Table 5:** 2 × 2 contingency table for exhaustion analyzed by the Maslach Burnout Inventory (MBI) or the Oldenburg Burnout Inventory (OBI).

MBI Exhaustion	OBI Exhaustion	
	Low	Medium/High
Low	43 (14.7)	26 (8.9)
Moderate/High	27 (9.2)	197 (67.2)

Values are expressed as N and percentage of the total sample.

**Table 6:** Comparison of scores obtained by the Maslach Burnout Inventory (MBI) and the Oldenburg Burnout Inventory (OBI) in participants stratified according to demographic and working characteristics.

	MBI			OBI		
	Exhaustion	Depersonalization	Gratification	Score	Exhaustion	Disengagement
**Age (years)**						
21–30 (69)	24.9 ± 12.4	8.74 ± 6.61	34.3 ± 7.08	2.41 ± 0.48	2.63 ± 0.52	2.19 ± 0.54
31–40 (85)	26.0 ± 13.1	9.86 ± 6.07	34.0 ± 7.33	2.41 ± 0.60	2.65 ± 0.68	2.16 ± 0.65
41–50 (75)	26.7 ± 12.1	7.57 ± 5.93	35.8 ± 6.85	2.35 ± 0.58	2.59 ± 0.64	2.11 ± 0.59
51–60 (61)	30.6 ± 13.8	9.18 ± 7.10	36.8 ± 5.52	2.39 ± 0.56	2.69 ± 0.63	2.09 ± 0.60
>60 (3)	32.0 ± 9.2	9.00 ± 10.8	42.3 ± 3.79	2.31 ± 0.47	2.67 ± 0.50	1.96 ± 0.94
**Sex**						
M (46)	23.0 ± 13.9 *	10.0 ± 7.10	36.2 ± 6.20	2.30 ± 0.61	2.51 ± 0.72	2.08 ± 0.59
F (247)	27.7 ± 12.6 *	8.93 ± 6.33	35.0 ± 6.98	2.41 ± 0.54	2.66 ± 0.60	2.15 ± 0.59
**Married**						
NO (126)	25.6 ± 13.1	9.05 ± 6.17	35.0 ± 6.70	2.38 ± 0.58	2.62 ± 0.66	2.13 ± 0.60
YES (156)	28.1 ± 12.6	9.25 ± 6.64	35.2 ± 7.04	2.40 ± 0.53	2.65 ± 0.58	2.15 ± 0.59
**Kids**						
NO (121)	27.1 ± 12.7	9.55 ± 6.45	34.6 ± 6.62	2.44 ± 0.51	2.69 ± 0.58	2.19 ± 0.55
YES (172)	26.8 ± 13.1	8.77 ± 6.46	35.6 ± 7.02	2.35 ± 0.58	2.60 ± 0.65	2.10 ± 0.62
**Chronic diseases**						
NO (217)	25.7 ± 13.0 **	9.13 ± 6.50	34.8 ± 7.22	2.38 ± 0.58	2.61 ± 0.64	2.15 ± 0.61
YES (76)	30.5 ± 11.8 **	8.99 ± 6.37	36.4 ± 5.63	2.42 ± 0.49	2.72 ± 0.57	2.12 ± 0.54
**COVID-19 department**						
YES (113)	28.3 ± 12.7	9.87 ± 6.28	34.1 ± 6.49 *	2.46 ± 0.55	2.73 ± 0.58	2.20 ± 0.62
NO (180)	26.1 ± 12.9	8.61 ± 6.54	35.9 ± 7.02 *	2.34 ± 0.56	2.58 ± 0.64	2.10 ± 0.57
**Emergency department**						
YES (81)	26.9 ± 13.0	9.74 ± 6.54	32.7 ± 6.62 ***	2.44 ± 0.59	2.69 ± 0.65	2.20 ± 0.65
NO (212)	27.0 ± 12.9	8.85 ± 6.43	36.2 ± 6.73 ***	2.37 ± 0.54	2.62 ± 0.61	2.12 ± 0.57
**Working years**						
1–5 (81)	24.0 ± 12.2 **	9.64 ± 6.60	34.3 ± 7.62	2.38 ± 0.52	2.61 ± 0.58	2.15 ± 0.54
6–10 (42)	29.5 ± 11.3 **	10.5 ± 5.36	33.6 ± 6.98	2.52 ± 0.46	2.74 ± 0.51	2.30 ± 0.59
11–15 (41)	23.9 ± 14.2 **	8.49 ± 7.02	34.9 ± 7.01	2.33 ± 0.65	2.58 ± 0.75	2.08 ± 0.60
16–20 (30)	24.5 ± 11.6 **	8.30 ± 6.82	35.8 ± 7.97	2.34 ± 0.64	2.56 ± 0.67	2.13 ± 0.67
>20 (99)	30.3 ± 13.1 **^,^^^	8.59 ± 6.41	36.5 ± 5.51	2.38 ± 0.56	2.66 ± 0.62	2.10 ± 0.59
**Infected by SARS-CoV-2**						
NO (252)	26.6 ± 13.0	8.79 ± 6.23 *	35.1 ± 6.93	2.38 ± 0.56	2.63 ± 0.63	2.13 ± 0.60
YES (41)	29.1 ± 12.1	11.0 ± 7.55 *	36.0 ± 6.46	2.43 ± 0.54	2.66 ± 0.60	2.20 ± 0.57
**Exposed to COVID-19 patients**						
NO (44)	28.2 ± 15.1	8.25 ± 6.35	36.3 ± 7.10	2.42 ± 0.61	2.63 ± 0.67	2.20 ± 0.61
YES (249)	26.7 ± 12.5	9.24 ± 6.48	35.0 ± 6.82	2.38 ± 0.55	2.64 ± 0.61	2.13 ± 0.59

Data are expressed as mean ± standard deviation. Statistical differences were assessed by independent student’s *t*-test or one-way analysis of variance. * = *p* < 0.05; ** = *p* < 0.01; *** = *p* < 0.001; ^^ = *p* < 0.01 vs. 1–5 at the post hoc analysis (Tukey test).

**Table 7:** Mediation analysis on the direct relationship between working factors and MBI domains of burnout.

Direct Effects			Estimate	Std. Error	z-Value	*p*	95% Confidence Interval	
							Lower	Upper
Support	→	MBI_Exhaustion	0.709	1.055	0.672	0.501	−1.358	2.776
PerceivedRisk	→	MBI_Exhaustion	−0.870	0.971	−0.896	0.370	−2.773	1.033
Workload	→	MBI_Exhaustion	4.667	1.016	4.595	<0.001	2.676	6.658
Social	→	MBI_Exhaustion	2.783	1.455	1.913	0.056	−0.069	5.634
ESupport	→	MBI_Exhaustion	2.568	1.084	2.369	0.018	0.443	4.694
Fatality	→	MBI_Exhaustion	2.578	1.169	2.206	0.027	0.287	4.869
PPE	→	MBI_Exhaustion	−1.400	1.196	−1.171	0.242	−3.744	0.943
Quit	→	MBI_Exhaustion	4.100	1.613	2.541	0.011	0.937	7.262
Support	→	MBI_Deperson	0.530	0.725	0.731	0.465	−0.891	1.952
PerceivedRisk	→	MBI_Deperson	−0.336	0.668	−0.503	0.615	−1.645	0.972
Workload	→	MBI_Deperson	1.262	0.699	1.806	0.071	−0.107	2.631
Social	→	MBI_Deperson	2.628	1.001	2.626	0.009	0.667	4.589
ESupport	→	MBI_Deperson	0.912	0.746	1.223	0.221	−0.549	2.374
Fatality	→	MBI_Deperson	1.239	0.804	1.542	0.123	−0.336	2.815
PPE	→	MBI_Deperson	−1.401	0.822	−1.703	0.088	−3.013	0.211
Quit	→	MBI_Deperson	0.710	1.110	0.640	0.522	−1.464	2.885
Support	→	MBI_Gratification	−0.015	0.762	−0.020	0.984	−1.508	1.478
PerceivedRisk	→	MBI_Gratification	−0.361	0.701	−0.514	0.607	−1.735	1.014
Workload	→	MBI_Gratification	−0.277	0.734	−0.377	0.706	−1.715	1.162
Social	→	MBI_Gratification	0.444	1.051	0.422	0.673	−1.616	2.504
ESupport	→	MBI_Gratification	−3.434	0.783	−4.384	<0.001	−4.969	−1.899
Fatality	→	MBI_Gratification	−0.072	0.844	−0.085	0.932	−1.727	1.583
PPE	→	MBI_Gratification	0.041	0.864	0.047	0.962	−1.652	1.734
Quit	→	MBI_Gratification	−0.375	1.166	−0.322	0.747	−2.660	1.909

*Note.* Delta method standard errors, normal theory confidence intervals, ML estimator.

## Data Availability

The data presented in this study are available on request from the corresponding author. The data are not publicly available due to privacy restrictions.
